# Development and Validation of Stability-Indicating Assay Method for a Novel Oxazolidinone (PH-192) with Anticonvulsant Activity by Using UHPLC-QToF-MS

**DOI:** 10.3390/molecules27031090

**Published:** 2022-02-06

**Authors:** Naser F. Al-Tannak, Oludotun A. Phillips, Husein J. Kamal, Ahmed Hemdan

**Affiliations:** 1 Department of Pharmaceutical Chemistry, Faculty of Pharmacy, Kuwait University, P.O. Box 24923, Safat 13110, Kuwait; oludotun.phillips@ku.edu.kw (O.A.P.); Husein.kamal@strath.ac.uk (H.J.K.); 2 Strathclyde Institute of Pharmacy and Biomedical Sciences, University of Strathclyde, Glasgow G1 1XQ, UK; 3 Department of Pharmaceutical Analytical Chemistry, Faculty of Pharmacy, Ahram Canadian University, Giza 12573, Egypt; hemmdan@yahoo.com

**Keywords:** antimycobacterial activity, linezolid, PH-189, substituted-glycinyl triazolyl-oxazolidinone, GC-MS, UPLC

## Abstract

The treatment of seizure disorders with currently available pharmacotherapeutic agents is not optimal due to the failure of some patients to respond, coupled with occurrences of side effects. There is therefore a need for research into the development of new chemical entities as potential anticonvulsant agents, which are different structurally from the existing class of drugs. We recently identified a novel triazolyl-oxazolidinone derivative, PH-192, as a potential anticonvulsant agent. PH-192 demonstrated protection comparable to phenytoin against both chemically- and electrically-induced seizures in rodents with little or no central nervous system side effects. However, PH-192 did not exhibit protection beyond 30 min; therefore, we decide to investigate a stability-indicating assay of PH-192 in plasma and other solutions. A reliable and validated analytical method was developed to investigate the stability of PH-192 for 90 min in human plasma, acidic, basic, and oxidative conditions, using a Waters Acquity ultra high-performance liquid chromatography (UHPLC) system with a quaternary Solvent Manager (H-Class). A simple extraction method indicated that PH-192 was stable in human plasma after 90 min at 37 °C, with more than 90% successfully recovered. Moreover, stress stability studies were performed, and degradants were identified using LC-QToF-MS under acidic, basic, and oxidative simulated conditions.

## 1. Introduction

Seizure disorders arise when brain nerve cells inappropriately and spontaneously fire action potentials, synchronously resulting in alterations in the sensory, motor, autonomic and psychological function of the afflicted individual. This manifestation of neuronal firing is called convulsion and the recurrent episodes of seizures result in a syndrome called epilepsy. Recurrent seizures significantly affect the quality of life and can sometimes be fatal if not adequately managed. Several anticonvulsant agents with different pharmacophores and chemical structures have been developed to control seizure disorders. However, effective, long-term control of this chronic condition with medication has not been very successful, with about 30% of the patients suffering from therapy-resistant epilepsy [1,2,3,4]. In addition, the use of most of these drugs is often accompanied by unwanted side effects. Therefore, there is considerable interest in the development of newer and more effective anticonvulsant agents that may cover several seizure types or treat currently untreatable seizures and with fewer side effects [5].

**Figure 1:**
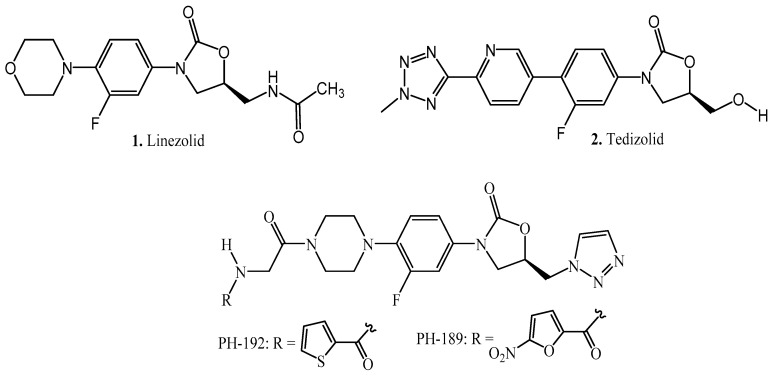
Chemical structures of oxazolidinones with antibacterial and anticonvulsant activities.

Oxazolidinone derivatives are exemplified by the presence of a five-membered heterocyclic ring system which has been identified as an important pharmacophoric group associated with the pharmacological properties of some clinically used drugs, namely, antibacterial, anticoagulant, and psychoactive agents [4,6,7,8,9,10]. Furthermore, systematic structural modification around the phenyl-oxazolidinone scaffold has yielded novel compounds with anticancer, antiepileptic, antithyroid, and more recently 5-lipoxygenase inhibitory properties [9,11,12,13,14,15]. The oxazolidinone-containing antibacterial agents, namely, linezolid 1 and tedizolid (2, Figure 1), have demonstrated clinical success with regards to their efficacy and safety profiles against susceptible and resistant Gram-positive cocci [16,17,18,19]. Based on these pharmacological properties, we previously reported the effects of a series of oxazolidinone derivatives on neuronal responses, which may correspond to their potential use as anticonvulsant agents with new pharmacophores [13,14,15]. Further structural modifications and in-vivo screening of selected derivatives’ anticonvulsant activity in rodents, using both electrically and chemically induced models of seizures, showed that some of the compounds protected rats and mice in all models without any observable anticonvulsant side effects. The most efficacious and safest of these compounds, PH-192, protected the animals only for 30 min and not beyond [15]. PH-192 [20], a thiophene glycinyl-oxazolidinone-containing derivative (Figure 1) produced a dose-dependent protection from seizures induced by using a 6 Hz stimulation protocol with an estimated ED50 of 34.5 mg/kg and 90 mg/kg in mice and rats, respectively. Furthermore, pretreatment of mice and rats for 30 min with 100 mg/kg of PH-192 for 30 min protected 75% and 66.6% of the animals, respectively, against 6 Hz-induced seizures. In addition, a 30 min IP pretreatment of rats with 100 mg/kg PH-192 protected 80% of the animals from seizure induced by pentylenetetrazole (PTZ) injection; this level of protection is comparable to that obtained for 40 mg/kg phenytoin, a reference antiepileptic drug that is clinically used for treating seizure disorders [15]. However, despite the favorable efficacy and safety profile of PH-192, the short duration of action (30 min) raised an important pharmacokinetic issue with this compound. Hence, prior to investigating the detailed pharmacokinetic behavior and brain distribution of PH-192, we decided to investigate an in vitro stability-indicating assay in plasma, acidic, and basic conditions. Our aim was to shed some light on the physicochemical stability properties of this compound.

We hereby report the development of a fast, reliable, and validated bio-analytical liquid chromatography–mass spectrometric (LC-MS) method to investigate the plasma concentration of PH-192 in the presence of other biological constituents, as well as in acidic, basic, and oxidative conditions to indicate any products of instability. Different instrumental analytical methods have been reported in the scientific literature for assessing the analysis of structurally diverse oxazolidinone derivatives and their instability products in plasma [21,22,23,24,25].

## 2. Results and Discussion

**Figure 2:**
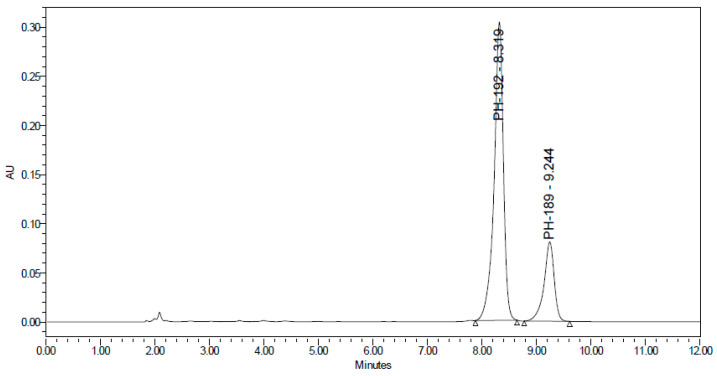
UHPLC-UV chromatogram of 40 µg/mL of PH-192 and 20 µg/mL of PH-189 as an internal standard.

As shown in Figure 2, the optimum resolution and peak shape were obtained with 0.1% *v*/*v* formic acid in water with acetonitrile (75:25 *v*/*v*) as a mobile phase. The flow rate was adjusted to 0.2 mL/min for better resolution and rapid separation.

### 2.1. Method Validation

#### 2.1.1. Linearity and Sensitivity

Linearity was achieved by plotting peak areas (y) versus the concentrations in the range of 1–80 μg/mL for PH-192 with correlation coefficients (r) ≥0.999. As for the calibration curve, performed in triplicate, the slopes and correlation coefficients showed high consistency, which demonstrated the reliability of the standard curve over the concentration ranges studied, as shown in Table 1. The LOQ was found to be 1 μg/mL for PH-192 with an RSD% of 5.8%. However, the LOD for PH-192 was found to be 0.33 μg/mL, using 10 μL as an injection volume, as shown in Table 1.

#### 2.1.2. Precision and Accuracy

In term of accuracy, the results were expressed as accuracy (%) of PH-192 in the samples. The overall results of PH-192 are demonstrated in Table 2, indicating the accuracy of the proposed UHPLC-UV method. With respect to precision, the values of the %RSD for intra-day and inter-day variations are given in Table 2. The % RSD values in both cases were found to be acceptable within a 2% limit, indicating that the developed method is repeatable.

#### 2.1.3. Evaluation of PH-192 Extraction and Stability in Human Plasma

The efficiency of the extraction method of PH-192 using liquid-liquid extraction was assessed by calculating the extraction recovery percentages. The extraction recovery was estimated from the peak areas of PH-192 in plasma and mobile phase. The extraction method was capable of extracting 94.38% of PH-192 from human plasma. PH-192 showed good stability in human plasma for 90 min at a temperature of 37 °C and no degradants were detected. The amount of PH-192 was calculated from the calibration curve equation and was found to be equivalent to 9.44 μg/mL out of 10 μg/mL of PH-192, which was spiked in the human plasma.

#### 2.1.4. Stability Study

**Figure 3:**
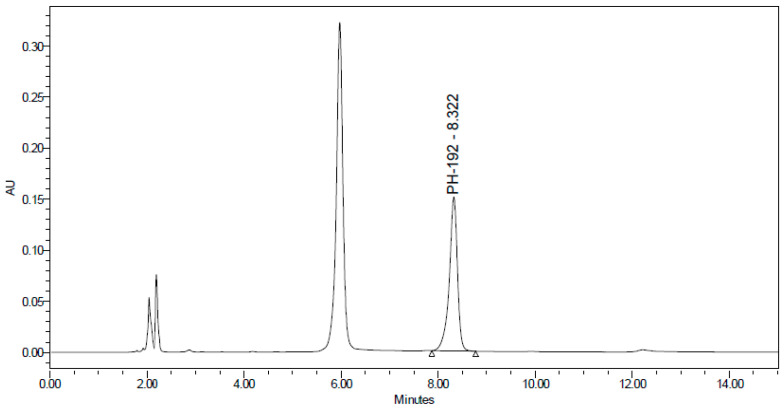
UHPLC-UV chromatogram for the basic degradation products of PH-192.

**Figure 4:**
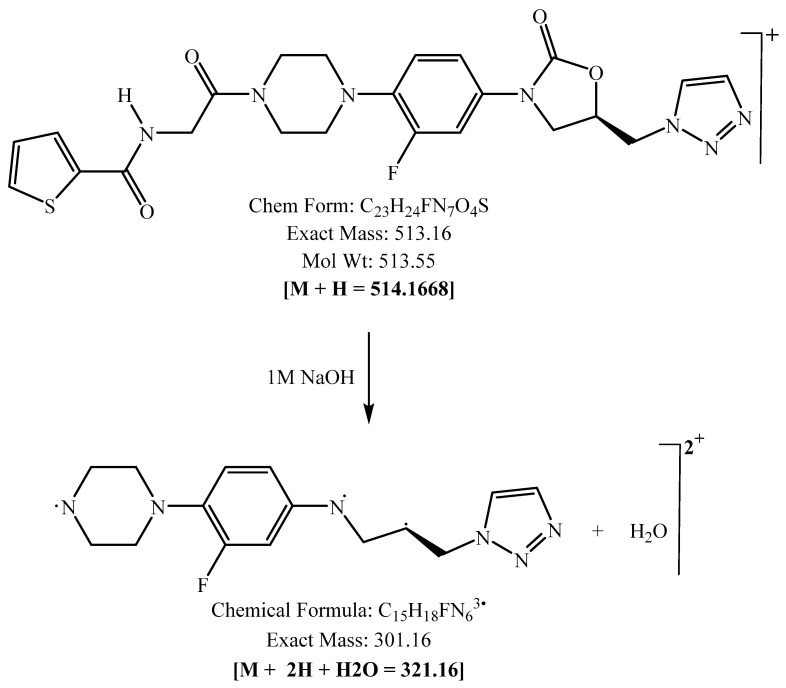
Degradation product of PH-192 after adding 1 N of NaOH.

**Figure 5:**
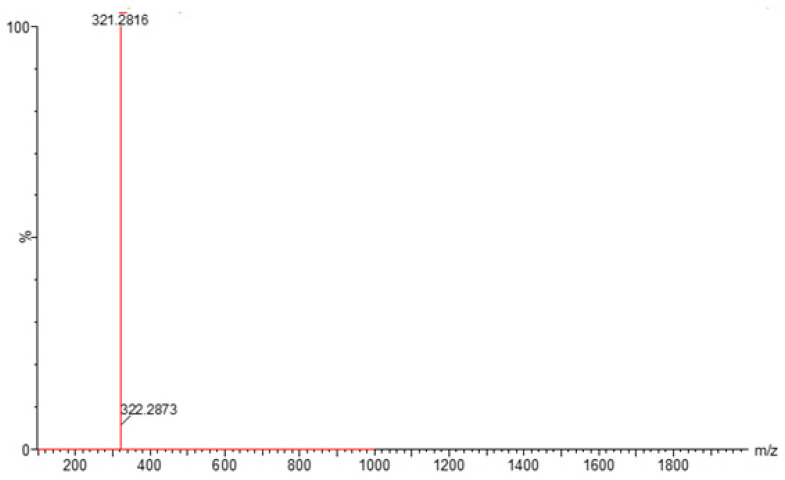
LC-QToF-MS analysis of PH-192 post-exposure to basic degradation.

**Figure 6:**
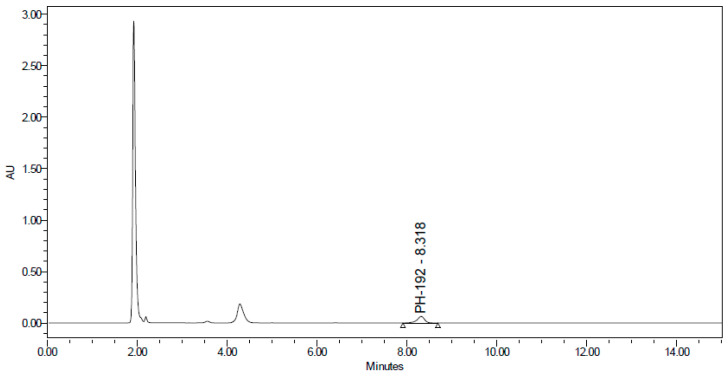
UHPLC-UV chromatogram for the oxidation degradation products of PH-192.

**Figure 7:**
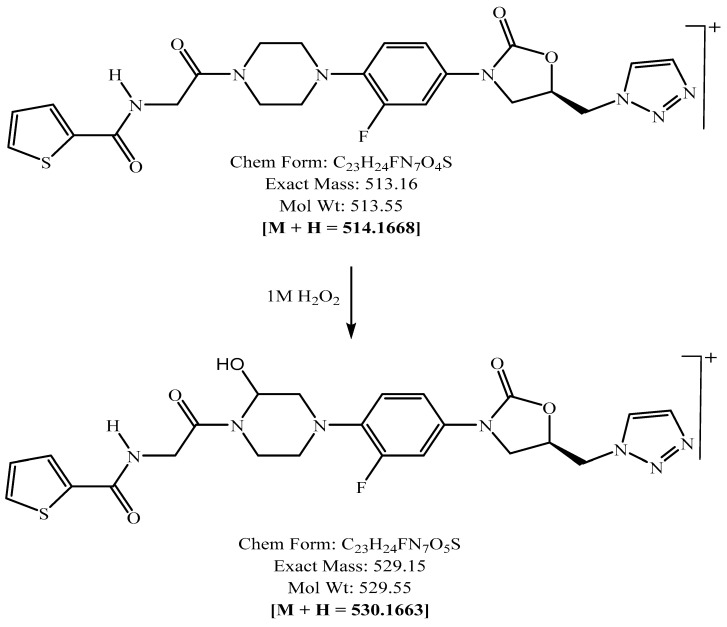
Degradation products of PH-192 after adding 1 N of H_2_O_2_.

**Figure 8:**
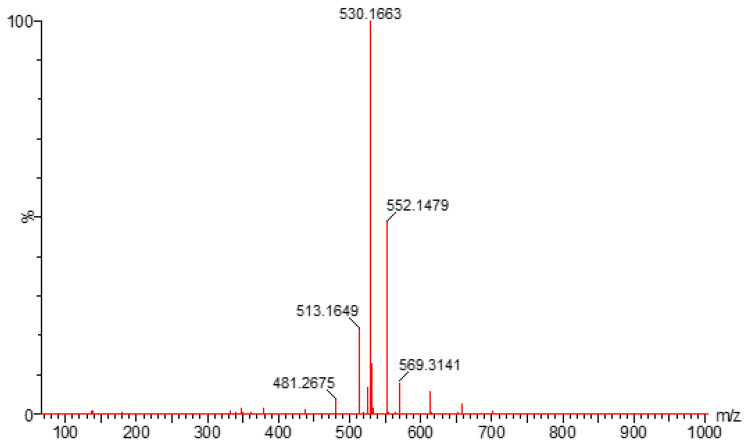
LC-QToF-MS analysis of PH-192 post-exposure to oxidation degradation.

PH-192 with a molecular weight of 513.5 g/mol was subjected to basic degradation by 1 N NaOH and heating at 90 °C for 90 min. There was one main degradation product detected via UHPLC-UV and was eluted around 5.9 min, as shown in Figure 3. The degradation product was identified and confirmed by LC-QToF-MS, as shown in Figure 3, Figure 4 and Figure 5. Moreover, PH-192 was exposed to oxidation by 1 N H_2_O_2_ and a main degradant product was detected by UHPLC-UV at 1.8 min as shown in Figure 6. LC-QToF was used to identify the degradation products, as shown in Figure 7 and Figure 8 [25]. On the other hand, PH-192 was found to be stable when subjected to 1 N HCl.

## 3. Materials and Methods

### 3.1. Chemicals

Drug-free human plasma was obtained from Kuwait Blood Bank, Al Jabriyah, Kuwait. HPLC-grade acetonitrile and other chemicals used in the adopted method were of analytical grade and obtained from Sigma Aldrich, Dorset, UK. “In-house” HPLC grade water was prepared with a MilliQ filter purchased from Millipore, Watford, UK. Syringe membrane filters (13 mm) were purchased from kinesis scientific expert, Cambridgeshire, UK. Nylon solvent filters (0.45 um) were used for solvent filtration and Water 20-positions.

### 3.2. Solutions

Stock standard solutions of PH-192 and the internal standard (PH-189) were prepared separately by dissolving 10 mg of the compounds in 10 mL of water:acetonitrile (75:25 *v*/*v*) to give stock concentrations of 1 mg/mL. Working solutions of PH-192 and the internal standard were prepared by diluting the stock solutions with water:acetonitrile (75:25 *v*/*v*) to obtain concentration of 500 μg/mL. Stock solutions were stable for at least 5 weeks when stored in a refrigerator (4 °C).

### 3.3. Human Plasma Extraction Procedure

Aliquots of 200 μL of human plasma were mixed with 10 μg/mL of PH-192. PH-192 was extracted by transferring 210 μL of human plasma to an Eppendorf tube, followed by the addition of 770 μL of acetonitrile (ACN) containing 20 µL of PH-189 as an internal standard and then vortexed. The samples were then centrifuged at 8000 revolutions per minute for 10 min. The supernatant was then collected into an HPLC vial as a final solution, ready for LC-MS analysis.

### 3.4. Instrumentation

#### 3.4.1. Ultra-Pressure Liquid Chromatography

Isocratic elution was carried out on a Waters Acquity UHPLC system with a quaternary Solvent Manager (H-Class), Sample Manager, and a UV detector, ACE C18 (50 × 3.0 mm, 3 µm) analytical columns were used for the analysis and method validation. Empower^®^ software was used for data processing and reporting. The mobile phase comprised filtered and degassed 0.1% formic acid in water and acetonitrile in proportion of 75:25 *v*/*v* and pumped at a flow rate of 0.2 mL/min. Column temperature was set to 30 °C and 10 μL of the sample was injected and analyzed at a wavelength of 254 nm.

#### 3.4.2. Liquid Chromatography-Mass Spectrometry

Waters^®^ Xevo G2-S QToF coupled with Waters^®^ Acquity UPLC system with binary Solvent Manager (I-Class) via the ESI interface. The operating parameters in the positive ion mode were as follows: the sheath gas and auxiliary flow rates were set at 30 and 5 (arbitrary units), respectively. The capillary voltage was set at 3.5 V, sampling cone was 55 V, and source temperature was 110 °C. Source temperature and desolvation temperature were set at 110 °C and 450 °C, respectively. Collision energy—2: 10 eV, 3: 15 eV, 4: 20 eV.

#### 3.4.3. Calibration Procedure for Mass Spectrometry

The calibration of mass spectrometry was achieved using an internal calibration procedure. Leucine enkephalin (*m*/*z* = 556.2771) was used as a reference compound and introduced to the ionization source at the same time as PH-192. As shown in Supplementary Materials Figure S1, the error was less than 1 ppm as the detected *m*/*z* for leucine encephalin was 556.2772. Sodium formate solution (0.5 µM solution) was used as a standard to calibrate the analyzer and MS/MS calibration to assess the accuracy and resolution, as shown in Figures S2 and S3.

### 3.5. Method Validation

Validation of the method was performed according to The International Council for Harmonisation (ICH) guidelines.

#### 3.5.1. Calibration Curve

Accurately measured aliquots of PH-192 were transferred from the working standard solution (1 mg/mL) into a series of 10 mL volumetric flasks and filled to volume with the mobile phase. The calibration samples consisted of five concentrations of PH-192 (1–80 µg/mL). The samples were injected separately into the ACE C18 column under a flow rate of 0.2 mL/min. The peak area of each drug was recorded against its concentration, the linearity of the curves was constructed, and regression equations were computed.

#### 3.5.2. Accuracy and Precision

The accuracy of the results was determined by calculating the accuracy (%) of three replicates of three different concentrations covering the linearity. The concentrations were calculated from the corresponding regression equations. The precision of the UHPLC-UV method for the combination was assessed by preparing six sets of PH-192 in the concentration ranges of the calibration curve. The precision of the UHPLC-UV method for PH-192 was estimated by preparing three different concentrations of pure standards of PH-192 (1, 20, and 80 μg/mL) within the linear range; samples were analyzed in triplicate, on a single day and three consecutive days, to calculate the intra-day repeatability and inter-day precision (intermediate precision) of the proposed method, respectively. A set (*n* = 3) was prepared at room temperature (22 °C), whereas five other sets (*n* = 3) were prepared and stored at 4 °C for mixtures dissolved in mobile phase samples for three days. The relative standard deviation percentage (%RSD) was used to calculate the intra- and inter-assay precision.

#### 3.5.3. Extraction Recovery and Matrix Effect

Two sets of standards containing 1, 20, 40, and 80 μg/mL of PH-192 were prepared. One set was prepared in human plasma and the other set in the mobile phase. The plasma standards were mixed with 20 μg/mL of internal standard and extracted as mentioned previously, whereas the standards of the other set were directly injected after mixing with internal standard (non-extracted samples). The extraction recoveries were calculated based on the slopes of the standard curve of PH-192 in the plasma and the mobile phase. Absolute recoveries of PH-192 and internal standard were also indicated by comparing the absolute values of the peak areas of PH-192 and internal standard in extracted and non-extracted samples.

#### 3.5.4. Evaluation of PH-192 Extraction and Stability in Human Plasma

A sample containing 20 μg/mL of PH-192 and 20 μg/mL of the internal standard were spiked in human plasma and placed in an oven for 90 min at 37 °C. PH-192 and the internal standard were extracted from plasma using liquid-liquid extraction and filtered by means of syringe membrane filters (13 mm) kinesis^®^.

#### 3.5.5. Limit of Detection (LOD) and Limit of Quantification (LOQ)

Stock solutions of PH-192 were prepared at concentrations of 1–100 µg/mL. The LOD and LOQ for PH-192 was determined at signal-to-noise ratios of 3:1 and 10:1, respectively.

### 3.6. Forced Degradation Studies

All stability tests were done on the active pharmaceutical ingredients and pharmaceutical formulations to simulate the actual conditions to which the dosage forms would be exposed during storage.

#### 3.6.1. Acidic Degradation

PH-192 (2 mg) was weighed and placed in a 4 mL vial. Two milliliters of 1 N HCl were added and heated at 90 °C for 90 min, allowed to cool down for 15 min, and then analyzed by means of LC-MS.

#### 3.6.2. Basic Degradation

PH-192 (2 mg) was placed in a 4 mL vial and subjected to basic stress conditions by adding 2 mL of 1 N NaOH. The sample was heated at 90 °C for 90 min, allowed to cool down for 15 min, and then analyzed by means of LC-MS.

#### 3.6.3. Oxidation Degradation

PH-192 (2 mg) was placed in a 4 mL vial and subjected to oxidative conditions by adding 2 mL of 1 N H_2_O_2_. The sample was then heated at 90 °C for 90 min, allowed to cool down for 15 min, and then analyzed by means of LC-MS.

## 4. Conclusions

Previous studies from our laboratory demonstrated that PH-192 (100 mg/kg) exhibited a time-dependent protection of rodents exposed to both chemically and electrically induced seizures with a peak effect at 30 min post-treatment and no observable protection at 2 h post-treatment. Thus, a fast, accurate, and reliable LC-ESI-QToF analytical method was established for the quantitation and stability indications of the novel oxazolidinone PH-192. This compound was found to be stable in acidic stress conditions after using 1 N HCl; however, in basic 1 N NaOH and oxidative 1 N hydrogen peroxide solutions it was found to be unstable. Moreover, compound PH-192 was found to be stable in human plasma for 90 min at 37 °C with a high percentage of recovery; therefore, it is a potent candidate that can be subjected to pharmacological studies in-vivo in animal anticonvulsant models.

## Figures and Tables

**Table 1:** Validation parameters of the proposed method.

Parameters	PH-192
Range µg/mL	1–80
Regression Equation	*y* = 0.0867x − 0.0403
Correlation coefficient (r)	0.9998
LOQ (µg/mL)	1
LOD (µg/mL)	0.33
Intra-assay precision ^b^	5.8
Inter-assay precision ^b^	7.4
Accuracy ^c^	96.66%

^b^ expressed as the RSD. ^c^ expressed as [mean % deviation = mean calculated concentra-tion/nominal concentration].

**Table 2:** Intra and inter- assay precision and accuracy data for PH-192 determination in bulk powder using UPLC-UV.

Precision	PH-192 Concentration μg/mL	Mean ± SD (*n* = 3) Observed/μg/mL	Precision ^a^ (%)	Accuracy ^b^ (%)
Intra-Assay Precision And Accuracy Data for PH-192 Determination in Bulk Powder Using UPLC-UV.	1	0.966 ± 0.056	5.8	96.66
	20	19.91 ± 0.158	0.8	99.55
	80	79.96 ± 0.165	0.2	99.96
Inter-Assay Precision And Accuracy Data for PH-192 Determination in Bulk Powder Using UPLC-UV.	1	0.943 ± 0.070	7.4	94.33
	20	19.77 ± 0.305	1.5	98.85
	80	79.27 ± 0.409	0.5	99.08

^a^ expressed as the RSD. ^b^ expressed as [mean % deviation = mean calculated concentration/nominal concentration ×100].

## Data Availability

Not applicable.

## Supplementary Materials

The following supporting information can be downloaded online. Figure S1: Spectrum for Leucine enkephalin as internal standard for mass spectrometry calibration; Figure S2: Mass Spectrometry accuracy assessment achieved using 0.5 µM sodium formate, Figure S3: Mass Spectrometry resolution assessment achieved using 0.5 µM sodium formate.

## Sample Availability

Samples of the compounds are not available from the authors.
