# Supplementary material for: Development and Validation of Stability-Indicating Assay Method for a Novel Oxazolidinone (PH-192) with Anticonvulsant Activity by Using UHPLC-QToF-MS
Source: Molecules. 2022 Feb 6;27(3):1090. doi: 10.3390/molecules27031090 (PMC8840153; doi:10.3390/molecules27031090)
Supplement: Supplementary file 1 [file molecules-27-01090-s001.zip › molecules-1557861-supplementary.pdf]

Supplementary Materials

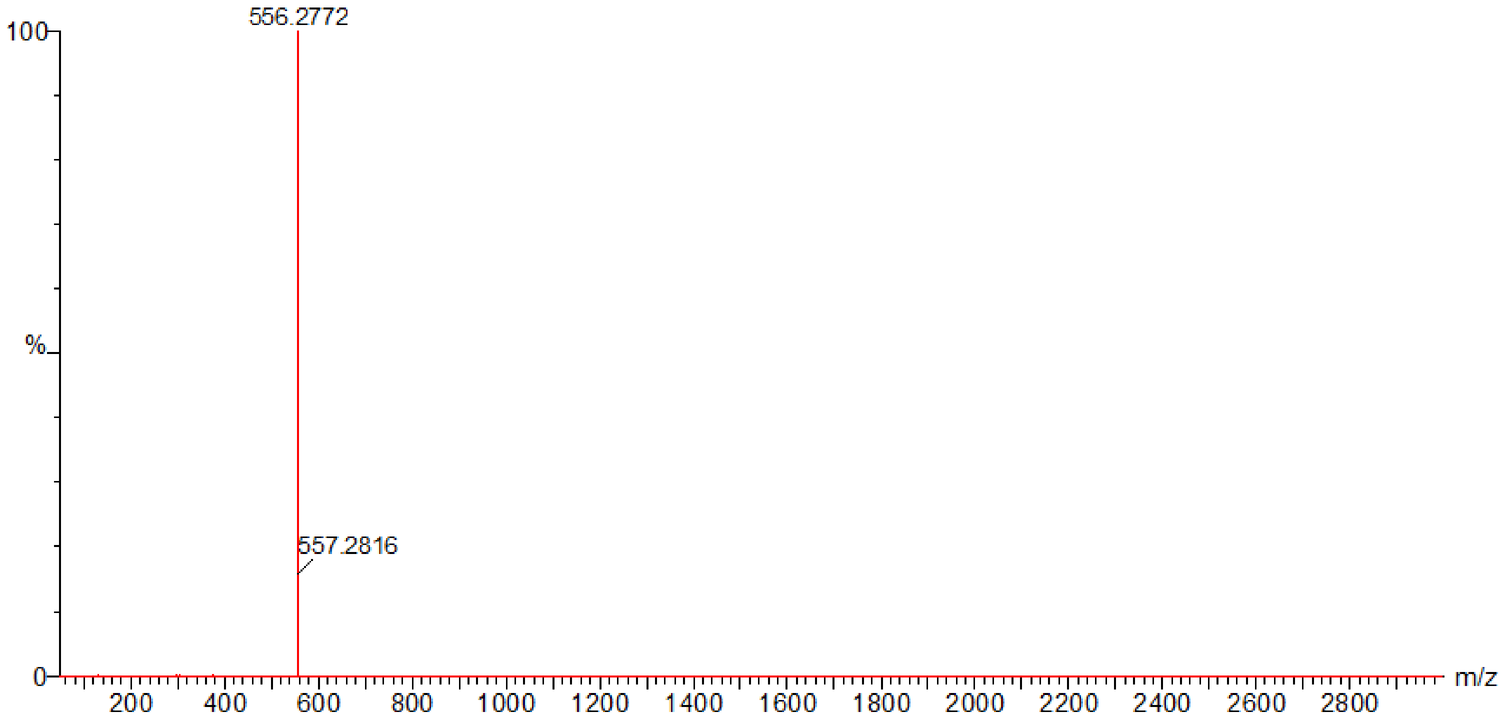

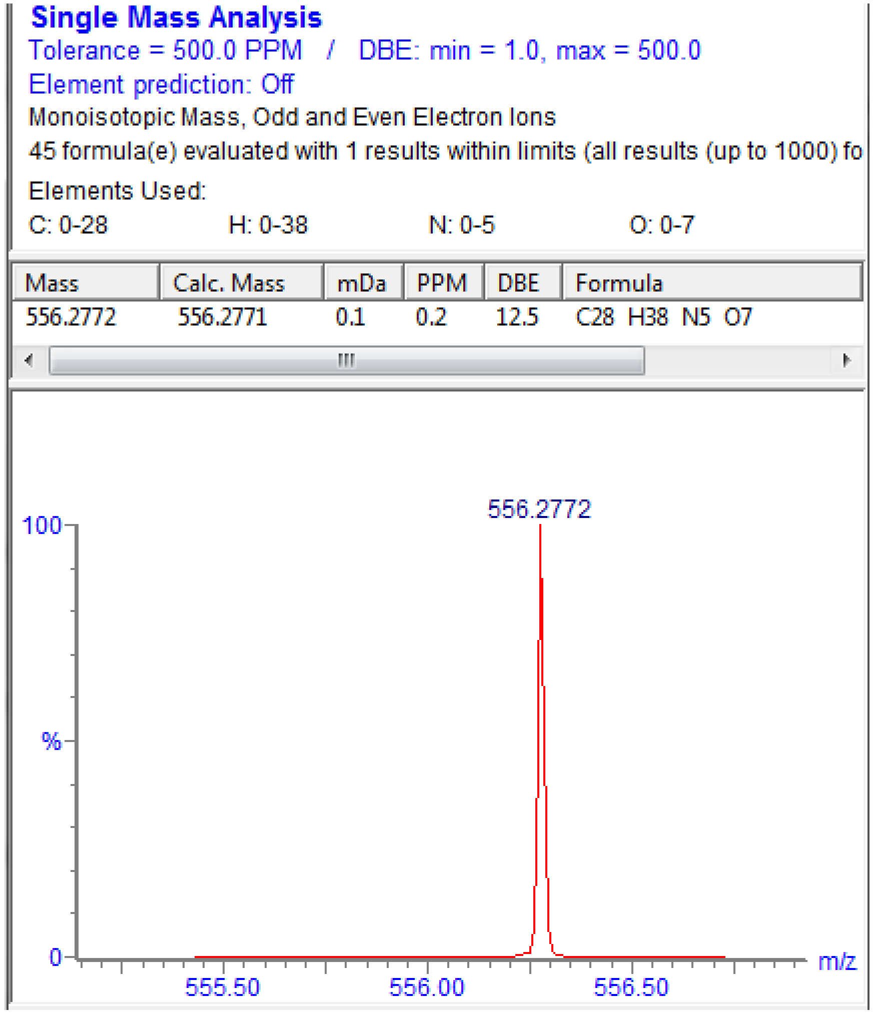

Figure S1. Spectrum for Leucine enkephalin as internal standard for mass spectrometry calibration.

Accept - Create Calibration (Assisted)

The calibration was accepted by the user.

Summary

RMS residual mass:	0.9 ppm (0.3 mDa)
95% confidence band:	1.0 ppm
Matched:	16 of 17 peaks

Action:	Create Calibration (Assisted)
Calibration Profile:	NaF_10JAN2021_50_1200_MS CAL
Mass Range:	50 to 1,200 Da
Mode:	Resolution
Polarity:	Positive
Flow Rate:	10.00
Calibration File:	C:\MassLynx\IntelliStart\Results\NaF_10JAN2021_50_1200_MS CAL-1.cal
Data File:	C:\MassLynx\IntelliStart.PRO\Data\NaF_10JAN2021_50_1200_MS CAL-2021-01-11-12-53-2.raw<br>Raw Data (0)
Reference File:	C:\MassLynx\Ref\ESI_NaFormate_Pos.ref (Sodium Formate)
Cone Voltage Used:	30.00
Capillary Voltage Used:	3.00

Instrument Id:	XEVO-G2SQTOF#NotSet
Computer Name:	MASSLYNX
Software Version:	MassLynx 4.1 SCN884
User Name:	waters

Data

Index	Reference mass	Measured mass	Intensity	Mass Resolution	Peak Width	Status
1	90.9772	91.1780	24,850	10,662	0.00860	✓ Pass
2	158.9646	159.1698	183,193	13,546	0.01170	✓ Pass
3	226.9520	227.1394	919,605	16,982	0.01340	✓ Pass
4	294.9395	Not Found	-	-	-	✗ Fail
5	362.9269	363.0461	416,969	20,659	0.01760	✓ Pass
6	430.9143	430.9897	895,931	22,334	0.01930	✓ Pass
7	498.9017	498.9282	592,390	22,973	0.02170	✓ Pass
8	566.8892	566.8627	742,282	24,242	0.02340	✓ Pass
9	634.8766	634.7939	810,278	25,418	0.02500	✓ Pass
10	702.8640	702.7222	902,266	26,456	0.02660	✓ Pass
11	770.8514	770.6487	708,241	27,321	0.02820	✓ Pass
12	838.8388	838.5729	697,228	28,016	0.02990	✓ Pass
13	906.8262	906.4958	476,374	29,010	0.03120	✓ Pass
14	974.8137	974.4172	400,608	28,684	0.03400	✓ Pass
15	1,042.8011	1,042.3370	311,947	30,254	0.03450	✓ Pass
16	1,110.7886	1,110.2551	222,529	30,741	0.03610	✓ Pass
17	1,178.7760	1,178.1725	167,802	29,265	0.04030	✓ Pass

17 Reference Peaks: 16 matched, 1 un-matched.

Calibration Chart:

Reference mass

974.8137

1,042.8011

1,110.7886

1,178.7760

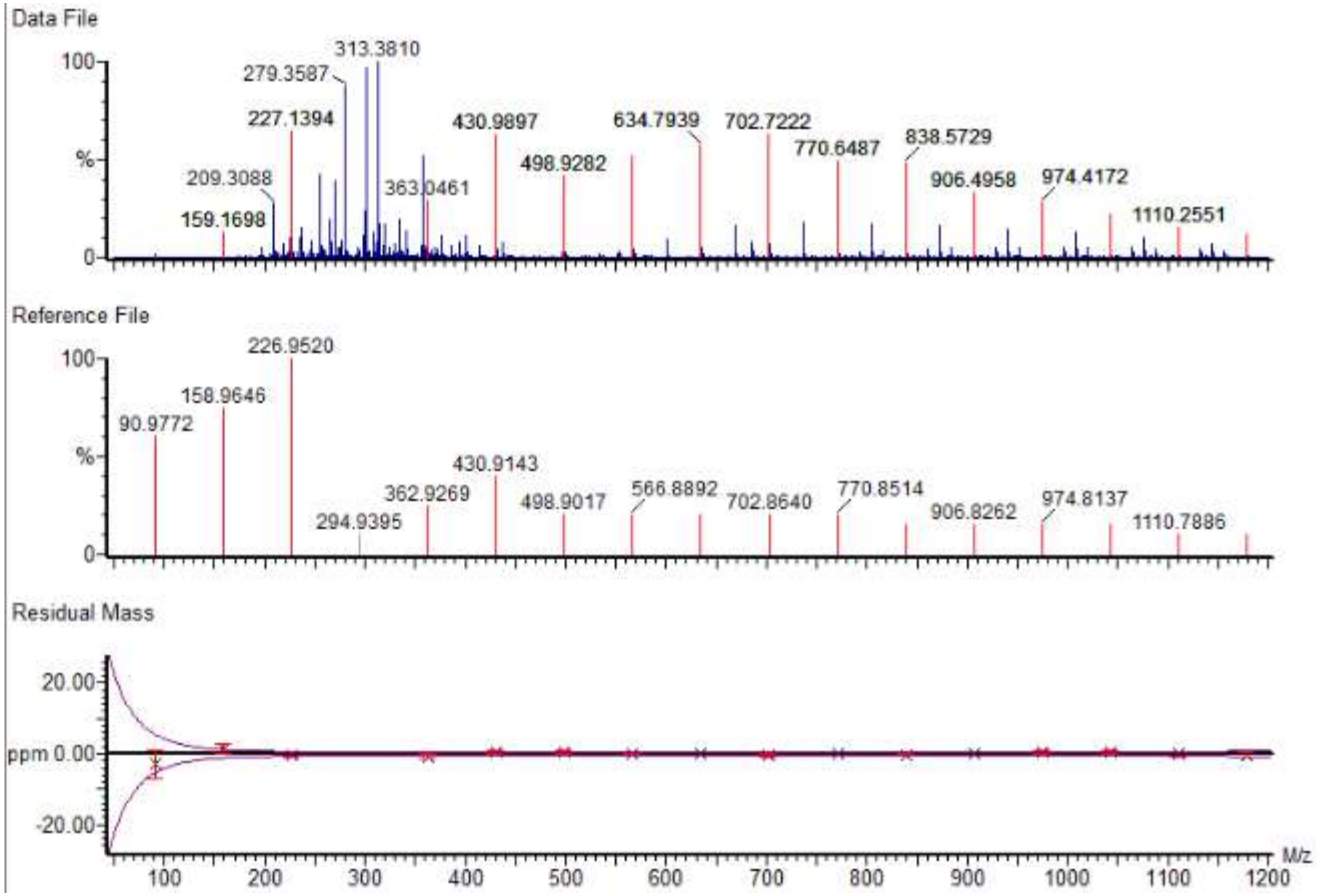

Figure S2. Mass Spectrometry accuracy assessment achieved using 0.5 µM sodium formate.

Results

Accept - Create Calibration (Assisted)

The calibration was accepted by the user.

Summary

RMS residual mass:	0.9 ppm (0.3 mDa)
95% confidence band:	1.0 ppm
Matched:	16 of 17 peaks

Action:	Create Calibration (Assisted)
Calibration Profile:	NaF_10JAN2021_50_1200_MS CAL
Mass Range:	50 to 1,200 Da
Mode:	Resolution
Polarity:	Positive
Flow Rate:	10.00
Calibration File:	C:\MassLynx\IntelliStart\Results\NaF_10JAN2021_50_1200_MS CAL-1.cal
Data File:	C:\MassLynx\IntelliStart.PRO\Data\NaF_10JAN2021_50_1200_MS CAL-2021-01-11-12-53-2.raw<br>Raw Data (0)
Reference File:	C:\MassLynx\Ref\ESI_NaFormate_Pos.ref (Sodium Formate)
Cone Voltage Used:	30.00
Capillary Voltage Used:	3.00

Instrument Id:	XEVO-G2SQTOF#NotSet
Computer Name:	MASSLYNX
Software Version:	MassLynx 4.1 SCN884
User Name:	waters

Data

Index	Reference mass	Measured mass	Intensity	Mass Resolution	Peak Width	Status
1	90.9772	91.1780	24,850	10,662	0.00860	✓ Pass
2	158.9646	159.1698	183,193	13,546	0.01170	✓ Pass
3	226.9520	227.1394	919,605	16,982	0.01340	✓ Pass
4	294.9395	Not Found	-	-	-	✗ Fail
5	362.9269	363.0461	416,969	20,659	0.01760	✓ Pass
6	430.9143	430.9897	895,931	22,334	0.01930	✓ Pass
7	498.9017	498.9282	592,390	22,973	0.02170	✓ Pass
8	566.8892	566.8627	742,282	24,242	0.02340	✓ Pass
9	634.8766	634.7939	810,278	25,418	0.02500	✓ Pass
10	702.8640	702.7222	902,266	26,456	0.02660	✓ Pass
11	770.8514	770.6487	708,241	27,321	0.02820	✓ Pass
12	838.8388	838.5729	697,228	28,016	0.02990	✓ Pass
13	906.8262	906.4958	476,374	29,010	0.03120	✓ Pass
14	974.8137	974.4172	400,608	28,684	0.03400	✓ Pass
15	1,042.8011	1,042.3370	311,947	30,254	0.03450	✓ Pass
16	1,110.7886	1,110.2551	222,529	30,741	0.03610	✓ Pass
17	1,178.7760	1,178.1725	167,802	29,265	0.04030	✓ Pass

17 Reference Peaks: 16 matched, 1 un-matched.

Calibration Chart:

Reference mass

974.8137

1,042.8011

1,110.7886

1,178.7760

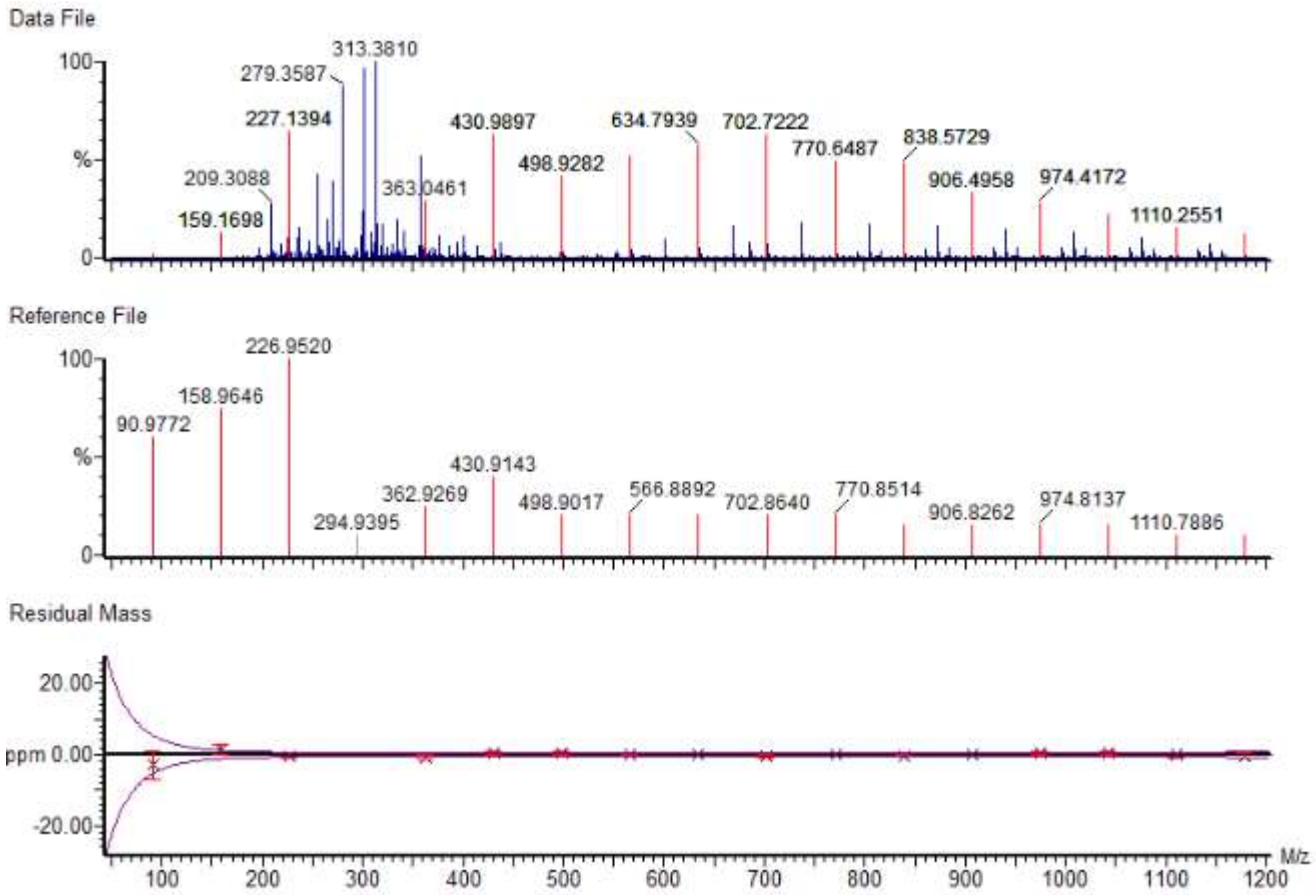

Figure S3. Mass Spectrometry resolution assessment achieved using 0.5 µM sodium formate.
